# Geofencing in location-based behavioral research: Methodology, challenges, and implementation

**DOI:** 10.3758/s13428-023-02213-2

**Published:** 2023-08-25

**Authors:** Yury Shevchenko, Ulf-Dietrich Reips

**Affiliations:** Research Methods, Assessment, and iScience; Department of Psychology; University of Konstanz, Universitätsstraße 10, Fach 31, 78464 Konstanz, Germany

**Keywords:** Geofencing, Internet-based research, Experience-sampling method, Mobile application, Notifications

## Abstract

This manuscript presents a novel geofencing method in behavioral research. Geofencing, built upon geolocation technology, constitutes virtual fences around specific locations. Every time a participant crosses the virtual border around the geofenced area, an event can be triggered on a smartphone, e.g., the participant may be asked to complete a survey. The geofencing method can alleviate the problems of constant location tracking, such as recording sensitive geolocation information and battery drain. In scenarios where locations for geofencing are determined by participants (e.g., home, workplace), no location data need to be transferred to the researcher, so this method can ensure privacy and anonymity. Given the widespread use of smartphones and mobile Internet, geofencing has become a feasible tool in studying human behavior and cognition outside of the laboratory. The method can help advance theoretical and applied psychological science at a new frontier of context-aware research. At the same time, there is a lack of guidance on how and when geofencing can be applied in research. This manuscript aims to fill the gap and ease the adoption of the geofencing method. We describe the current challenges and implementations in geofencing and present three empirical studies in which we evaluated the geofencing method using the *Samply* application, a tool for mobile experience sampling research. The studies show that sensitivity and precision of geofencing were affected by the type of event, location radius, environment, operating system, and user behavior. Potential implications and recommendations for behavioral research are discussed.

Nowadays, with technological development of the mobile Internet, research is no longer limited to the laboratory, desktop computers, or bulky laptops, but can be conducted anywhere the Internet can reach via smartphones and other small devices. In support of this tendency that goes beyond Web-based research (e.g., Reips, 2008, 2021), scientific investigation has been done increasingly via participants’ smartphones in recent years (Pinter et al., 2015), and the number of mobile health (mHealth) applications is growing (Agarwal et al., 2016). The expansion of mobile Internet coverage and the fact that people carry smartphones everywhere make it possible to study people’s experiences across spatial boundaries and in the midst of activities (Klein & Reips, 2017). The ability to take into account participants’ locations is important because many experiences are defined by happening in specific places, such as shopping (in a store) or visiting the ill (in a hospital). Experiences may be changed or shaped by the location where they happen, e.g., singing at a concert hall versus a church. Memory has been shown to be prone to biases and distortions (Shiffman et al., 2008), therefore an ideal time to survey participants is when they are at or have just left a specific location, like, e.g., in exit polls during elections.

Geofencing technology enables location-aware research by defining a virtual border around a geographic area. A smartphone can detect incidents of entering or leaving the geofenced area, and each of these events can trigger an action, which depends on the application that uses geofencing. As we will demonstrate in this article, one such event can be sending a link to a mobile survey, where a participant is asked about the current experience. Other possible applications of the method include the presentation of any study materials that can be put on a website linked to the notification: e.g., interventions or experimental tasks.

Geofencing is based on the geotracking functionality that allows a mobile device to determine its location. Historically, the first mobile location-based services developed in 2000–2007 derived the user’s position from the coordinates of the serving base station, i.e., the Cell-ID protocol (Willaredt, 2011). The use of this technology was limited to point-of-interest (PoI) services, such as finding a nearby restaurant. Later, the Global Positioning System (GPS) was developed and became a standard feature on most smartphones. Some limitations of GPS, such as low signal quality indoors or near large buildings, long acquisition time to obtain the first position, and high battery consumption, have been addressed by the development of hybrid positioning systems, which are a combination of GPS, Wi-Fi, and Cell-ID positioning. For example, using the Secure User Plane (SUPL) protocol, the user’s device first obtains the position via Wi-Fi and cell identifiers, and then a more accurate GPS protocol is employed using GPS satellites. Further technological advancement enabled background tracking, which runs as a background process on a smartphone (Küpper et al., 2011). Beyond reliable and valid measures of longitude and latitude, Stieger and Reips (2019) showed that GPS sensors in smartphones can also provide valid measures of altitude.

Geofencing is different from geotracking, which continuously records a user’s absolute geographic location at regular intervals, depending on the app’s settings (e.g., Geyer et al., 2019). The geotracking method has been widely used in smartphone-based research, but it entails problems such as missing data (Bähr et al., 2020), potential violation of user privacy, and users’ concerns about battery drain (Liss et al., 2018). Location tracking technologies have been criticized for their intrusion into private life, sometimes disguised under the mask of security (Zuboff, 2019). Compared to geotracking, geofencing can be perceived as less intrusive. For example, if the location of interest is the participant’s home, using geofencing will not expose the home address, but researchers can still send notifications and conduct surveys when people leave or enter the home. Geofencing also allows the smartphone to apply strategies for energy-efficient tracking, as there are algorithms that do not require continuous geotracking (Nakagawa, 2013). Küpper et al. (2011) described possible methods, such as checking the location less frequently or using the built-in accelerometer to activate tracking only during movements. Finally, the smartphone can also use approximate methods, such as Cell-ID protocol, as a first step to determine if a more accurate geotracking method should be used afterwards.

The geofencing method can trigger an event in a mobile application only when some predefined geographical borders are crossed. Moreover, when using geofencing, the absolute coordinates of a participant’s location do not need to be sent to the server as geofencing can be handled by the participant’s smartphone. This is ideal for situations where a researcher studies a sensitive topic and must not collect location data for privacy reasons. This also offsets the burden of handling sensitive private data for a researcher, such as anonymization of location data (Attwood et al., 2017). In addition, geofencing eliminates the need to analyze large-scale multidimensional geotracking data that might consist of thousands of timestamps with coordinates and requires preprocessing and aggregation.

## Previous research using geofencing

Because the technique itself is relatively new, research using geofencing has only begun in the last decade. To our knowledge, this article is the first systematic description of the geofencing method and its evaluation in a behavioral research journal. The method has the potential to generate novel theoretical perspectives and practical applications.

Geofencing can provide insights for the ecological approach that studies the structure of contextualized social interactions, the link between knowledge and action, and the situated nature of knowledge (Good, 2007). The use of geofencing is in line with a theoretical shift from studying the psychology of people passively perceiving the environment to the psychology of people acting in the current situation. In social and personality psychology, geofencing can be a valuable tool for studying person–situation interactions. The method can help to characterize individual differences in perception of situations (Rauthmann et al., 2014; Ziegler et al., 2019) and behavior across different locations (e.g., “if...then...” situation–behavior relations in Mischel & Shoda, 1995). Geofencing can benefit research that combines psychological theories with digital movement data. For example, surveys conducted at specific locations, such as sports events or segregated neighborhoods, can explore the sense of identity, group belonging, or perceptions of out-groups (for other examples, see Hinds et al., 2022). Such studies can inform social identity and intergroup contact theories. Researchers can use publicly available databases or maps that provide location information to select potential places of interest and specify survey questions (e.g., the *Atlas of Inequality* in Moro, 2019).

Previous research underscored that geofencing can improve understanding of behavior in context, with an emphasis on potential applications. Mair et al. (2019) described the importance of incorporating neighborhood and regional contexts into research on alcohol use by combining risk assessments of exposures within specific locations. Risk assessment models can be further updated based on real-time data to generate interventions tailored to a user and delivered at a time of high risk for a lapse (Forman et al., 2019). This makes geofencing a viable tool for clinical and mobile health research. Mobile health research can benefit from including locations in the analysis, as many addictive behaviors, such as alcohol or nicotine consumption, are triggered by specific places. Potential applications for location-based interventions include a number of health issues, such as physical activity, alcohol use, smoking, obesity, and mental illnesses (in Nahum-Shani et al., 2018). Attwood et al. (2017) used geofencing as part of an alcohol reduction intervention. The mobile app sent messages when participants were at locations where they needed support to regulate alcohol consumption. Wray et al. (2019) examined social and environmental factors of alcohol use by sending surveys to participants at places associated with heavy drinking. About 40% of surveys arrived when participants were not at the intended location, indicating that geofencing technology is still in development. On the other hand, Naughton et al. (2016) concluded that geofencing was reliable and accurate in identifying smoking locations. The authors developed a context-triggered lapse-prevention system, in which supportive messages were sent to smokers who had decided to quit when they were entering the areas associated with smoking. Besoain et al. (2020) used a co-design approach to develop a mobile application UBESAFE that informed users about the potential risk of HIV near areas with a high probability of sexual encounters. An interesting feature of this app was that users could add their own health-promoting messages and share the areas with a community. Coral et al. (2020) proposed using geofences around gambling establishments to discourage their attendance. The authors developed a mobile app that could apply geofencing even if the GPS was turned off. Nguyen et al. (2017) showed the usefulness of geofencing in ascertaining hospitalizations; however the accuracy of geofencing validated by medical records was moderate. Other geofencing applications include dental clinic promotion (Wright et al., 2021), employment research by geofencing at job centers (Haas et al., 2020), and using geofencing for disaster information systems (Suyama & Inoue, 2016). During the COVID-19 pandemic, some mobile applications, such as the *luca App* developed in Germany (culture4life GmbH, 2021), used geofencing to automatically check people out when they left a location.

Overall, previous research has shown that geofencing can be applied for data collection in the field (see the summary of the existing and potential research applications for geofencing in Table 1). However, the geofencing method has not been systematically evaluated in terms of its sensitivity and precision. With the present manuscript we intend to fill this gap: we report three experimental studies in which we tested the method. Thus, this manuscript can help researchers understand the influence of various factors, such as the type of operating system or location radius, and plan a geofencing study in their research area. Second, we provide the implementation of the geofencing method with a mobile application that allows researchers to conduct their own geofencing studies. In the following, we discuss this implementation in detail.

**Table 1:** Existing and potential applications of geofencing in research

Research area	Research topic or application	Locations	Relevant research or software
Addiction research and risky behavior	Alcohol use	Local bars, supermarkets	Mair et al. (2019) Attwood et al. (2017) Wray et al. (2019)
	Smoking	Areas associated with smoking	Naughton et al. (2016)
	Sexual behavior	Places with a high probability of sexual encounter	Besoain et al. (2020)
	Gambling	Gambling establishments (e.g., casino)	Coral et al. (2020)
Industrial and organizational psychology	Employment research	Job centers	Haas et al. (2020)
	Monitoring work hours	Work facility (e.g., medical center)	Owei et al. (2021)
Public health and public safety	Ascertaining hospitalizations	Hospitals	Nguyen et al. (2017)
	Disaster information systems	Dangerous areas	Suyama & Inoue (2016)
	Warning people about crime activity	Crime-prone areas	Mane et al. (2021)
	Contact tracking	Places of people gathering (e.g., restaurant, party, family meeting)	*luca App,* culture4life GmbH (2021)
New potential applications			
Health behavior	Dietary behavior	Supermarkets, shopping malls, fast-food courts	
	Doctor visit	Doctor’s office, clinic	
	Exercise	Gym, swimming pool	
Animal behavior	Dog/cat behavior	House and adjacent areas	
Urban studies	Perception and interaction with the environment	Public areas, such as downtown, museums, and parks	

## Implementation of geofencing in *Samply*

Geofencing itself is performed by the smartphone’s operating system. To use geofencing, application developers utilize the software libraries provided by *LocationManager Class* in Android (Android, 2023) and *Core Location Framework* in iOS (Apple, 2023). Researchers must either program a mobile application from scratch or use one of the available applications.

In the example implementations described in this article, we will be using the software *Samply*, which was developed in our iScience research group (Shevchenko et al., 2021). Other researchers can also freely use *Samply*, without having to program an app or set up a server. *Samply* supports experience-sampling studies on mobile devices and consists of the *Samply* website for researchers (https://samply.uni-konstanz.de) and the *Samply Research* mobile app for participants. Participants can download the app at no cost from the respective Apple or Google app stores^1^. Researchers can create and send push notifications containing links to online surveys or other websites^2^. Once the participant taps on the notification, the link opens in a mobile web browser. *Samply* provides schedule-based notifications that allow participants to be notified at certain predefined or random times or at time intervals (Shevchenko & Reips, 2022).

**Fig. 1:**
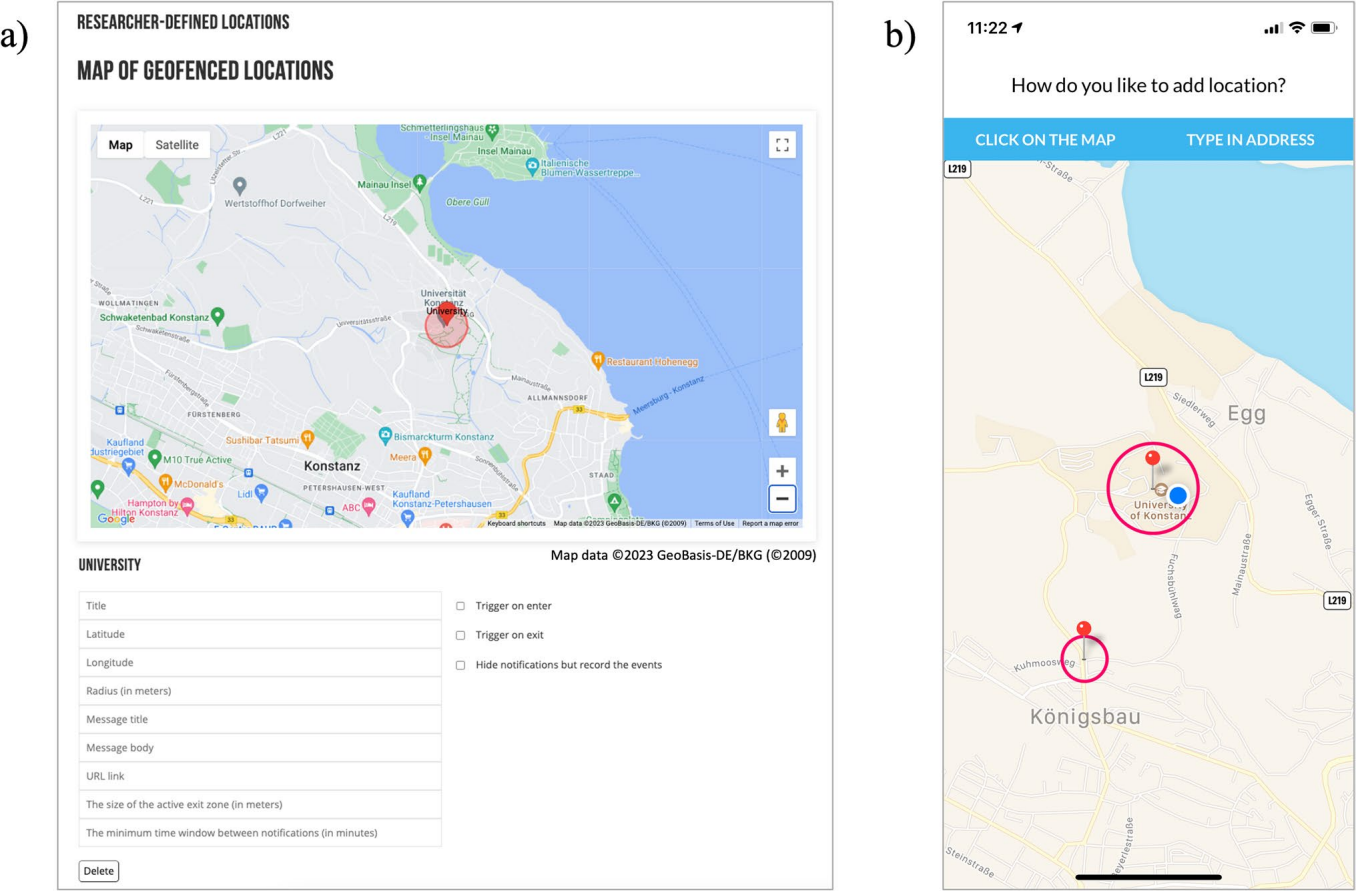
Geofencing interface on the *Samply* website for researchers (**a**) and mobile application for participants (**b**)

In the current work, we extended the *Samply* website and mobile application code and built a user-friendly geofencing interface to create and edit geofenced locations on the website for researchers and in the mobile application for participants. Thus, *Samply* allows both researchers and participants to customize locations, which broadens the software’s application scope – a benefit for software (Rough & Quigley, 2020). The events of entering or leaving a location can trigger sending a notification with a web link to the participant. For the locations determined by a researcher, the *Samply* website requires entering GPS coordinates and defining the radius of the fence. The coordinates, longitude, and latitude can be found in publicly available maps, e.g., Google Maps, Apple Maps, Microsoft Bing Maps, or OpenStreetMaps. Alternatively, locations can be selected through the drag-and-drop map interface (see Fig. 1a). *Researcher-defined locations* can be the same areas for all participants, such as a university or a city center. *Participant-defined locations* are unique to each participant, such as home or workplace. Participants use the mobile app interface to select locations on the map (see Fig. 1b). The coordinates are stored locally on the smartphone and not shared with the *Samply* website or the researcher to protect participants’ privacy. While the operating system continuously monitors the location in the background, the GPS data is neither stored on the smartphone nor shared with the server.

## Factors influencing geofencing

A number of factors can affect the accuracy of geofencing: radius of the geofence, type of mobile operating system and device (Kuhlmann et al., 2020), Wi-Fi access, and type of geofencing event. The common shape of the geofenced area is a circle that is defined by its radius. Although there are possibilities of defining the boundaries in the polygon shape, this functionality is not equally supported in iOS and Android devices. Regarding the radius, previous research has used different sizes, e.g., 10 to 30 m by Wray et al. (2019) or 100 m by Naughton et al. (2016). A radius that is too small can increase the number of misses and reduce the accuracy of geofencing.

The way a smartphone responds to geofencing events depends on the type of mobile operating system – almost all smartphones run either iOS or Android^3^. The iOS documentation specifies 10 m as the smallest possible radius^4^, although anecdotal evidence from Internet forums suggests that the use of the 10-m radius could be problematic. Since iOS14, released in the fall of 2020, there are two types of user location available to applications: precise and approximate. Geofencing requires a precise location and that a user grants permission always to access the location. To decrease the likelihood of a false alarm, the iOS waits for a user to travel a minimum distance over the geofenced area and remain on the same side of the geofence for at least 20 s^5^.

The Android documentation recommends that the minimum geofence radius be between 100 and 150 m for best results^6^. Beginning with Android 12, released in the fall of 2021, there is also a distinction between precise and approximate user locations available to an application. With enabled Wi-Fi (even if the smartphone is not connected to a Wi-Fi network), the minimum radius can be between 20 and 50 m. If an indoor positioning system is available, the radius can be as small as 5 m. When Wi-Fi is disabled, the smartphone relies on GPS or the cellular network to determine the location^7^. The lack of reliable network connection or the latency of location queries can also affect the accuracy of geofencing. An Android smartphone usually requests the current location every second minute. If the device has been stationary for a significant amount of time, the latency may increase up to 6 min.

The way Android and iOS operating systems implement geofencing represents different approaches to maximizing the efficiency of geofencing without putting too much strain on the smartphone battery. In summary, iOS relies more on distance measures, while Android reduces the frequency of geotracking. These differences may become important for real-world applications, therefore the comparison of the two operating systems was included in our empirical studies.

With regard to similarities between the operating systems, geofencing in both systems still works in the background even when a user closes the mobile application that uses geofencing. The system continues to monitor the registered regions and launches the application in case of an event. Both operating systems impose a limit on the number of geofences that a user can create: 20 for iOS and 100 for Android. When a user uninstalls the mobile application that uses geofencing, the geofencing service is stopped. For iOS, a restart of geofencing in the application is required if the user has disabled Background App Refresh (either for the app or for all apps). For Android, a restart is necessary if the user has rebooted the smartphone, cleared the app’s data, cleared Google Play services data, or disabled Android’s Network Location Provider. Researchers should thus provide instructions on how to restart geofencing in case participants accidentally disable it (e.g., by rebooting the smartphone).

Previous research reported problems with detecting geofencing events when Wi-Fi was disabled in iOS (Suyama & Inoue, 2016), so we included a test between smartphones with enabled and disabled Wi-Fi in Study 1. In addition, Suyama and Inoue (2016) found differences in the distance to the fence at which users were notified for entering and exiting events in iOS: While the distance for enter events was 20 to 30 m, it was 120 to 130 m for exit events in their study. This motivated us to systematically observe the differences between enter and exit events in our studies.

To evaluate the feasibility of the geofencing implementation, we conducted three empirical studies. By conducting these empirical studies and with this manuscript we aim to address the lack of methodological, technical, and practical guidance on geofencing in behavioral research and help researchers understand the influence of various factors on the results of the method’s application.

## General method

To evaluate the geofencing method and its implementation in Samply, we analyzed its performance using sensitivity, precision, distance, and time measures. Sensitivity and precision are measures constructed from a confusion matrix (see Table 2). Sensitivity is the probability of receiving the notification given that its corresponding event has occurred. Sensitivity can be calculated as the proportion of correct hits to the sum of correct hits and misses. Precision shows how accurate was the notification given that the notification was sent. Precision can be calculated as the proportion of correct hits to the sum of correct hits and false alarms. The distance and time measures refer to the absolute distance and time difference, respectively, between the location where the notification was received and the geofenced area.

**Table 2:** Confusion matrix for geofencing events

		Actual event	
		The participant has entered/left the area	The participant has not entered/left the area
Detected event	Notification was sent	Correct hit	False alarm
	Notification was not sent	Miss	Correct rejection

We used different methods and measures in our three empirical studies (see Table 3). The evaluations in Study 1 and Study 2 were conducted by the first author and research assistants, while Study 3 involved naïve participants. In Study 1, we recorded the location of geofencing events using the smartphone GPS tracker available via the mobile Internet browser. The main measures were the geofencing sensitivity and the distance between the center of the geofenced area and the location where the notification was received. In Study 2, we replicated and validated the results from Study 1 by using an external GPS tracker to record the location and timestamp of geofencing events. Study 2 measured sensitivity, the distance between the fence and the location where the notification was received, and the time difference between the two. In both Study 2 and Study 3, sensitivity was modelled as a binary variable (whether or not the notification was received given that the evaluator was at the location), which allowed us to assess the effect of each factor. In Study 3, students at the University of Konstanz received notifications with an online survey when they entered or left the university. Sensitivity and precision were computed based on the number of received notifications for each participant. The data and analysis scripts are available at OSF (https://osf.io/mg6f4/).

**Table 3:** Overview of studies

	Study 1	Study 2	Study 3
Participants	Evaluators (*N* = 4)	Evaluators (*N* = 2)	Students (*N* = 58)
Number of tests	360	120	-
Independent variables	Event type, OS, radius, area, Wi-Fi	Event type, OS, radius, user behavior	Event type, OS
Dependent variables	Sensitivity (a notification was received or not) Distance to the center	Sensitivity (a notification was received or not) Distance and time difference with the fence	Sensitivity (the number of received notifications) Precision
Data collection method	Evaluators and the smartphone’s web browser recorded locations	Evaluators and an external GPS tracker recorded locations and timestamps	Students entered the data in an online survey
Time	2021, July–November	2022, July–August	2022, November–December

## Study 1

The goal of Study 1 was to assess and calculate sensitivity and the distance to the center in different conditions using the absolute geolocation of testers as a benchmark.

### Method

#### Participants

We evaluated the geofencing method in a series of empirical tests, which were performed by the first author and research assistants.

#### Procedure

**Fig. 2:**
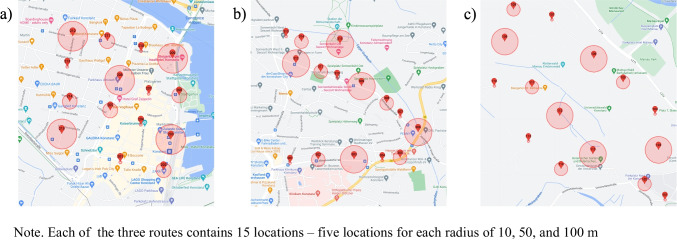
Routes with geofenced locations in downtown (**a**) residential area (**b**), and forest (**c**). *Note*. Each of the three routes contains 15 locations – five locations for each radius of 10, 50, and 100 m

Four evaluators walked with their smartphones along a pre-defined route in three different environments: downtown, residential area, and forest (see Fig. 2). These three environments were selected because they represent areas with a wide variety of structures (e.g., buildings, Wi-Fi networks) and available Internet connectivity, which might affect network connectivity and therefore geofencing. The downtown area included locations in the city center characterized by a high density of commercial buildings such as shops and restaurants. The residential area was composed of a mixture of single-family houses and apartment buildings. The forest was a dense collection of trees outside the city. Additionally, we systematically varied the factors such as the type of geofencing event (enter or exit), type of OS (Android, iOS), and Wi-Fi access (turned on or off). Therefore, the testers completed a route in each environment eight times in a fully crossed 2 x 2 x 2 design with two event types, two operating systems, and two Wi-Fi conditions. To evaluate the effect of the geofence radius, there were 15 locations for each route – five locations for each radius of 10, 50, and 100 m (see Fig. 2). In total, there were 360 tests performed (3 environments x 8 repetitions x 15 locations).

During the tests, the evaluators walked along the pathways that went through predefined geofenced areas. They were instructed to carry the smartphone conveniently in their dominant hand and not to use it for other purposes (e.g., texting or web browsing). By carrying the smartphone in the hand, we wanted to minimize the probability of missing the notification if the smartphone was in the pocket or the bag and the evaluators missed the sound or vibration. When they heard the signal of notification arrived or/and felt vibration of the smartphone, they stopped moving, attended to their smartphone, and clicked on the notification, which opened a mobile browser with a one-question survey about the current location. At this moment, the absolute geolocation of the user was automatically recorded in the smartphone’s mobile browser. After answering the survey question, the evaluators closed the browser app and continued moving.

We measured the geofencing sensitivity by recording whether the notification was sent for every location the evaluator visited. We also computed the distance between the actual location of the smartphone at the moment of answering the survey question and the center of the geofenced area.

#### Materials

##### Smartphones

The four evaluators used their own smartphones: iPhone 11, iPhone 12, Xiaomi Redmi Note 10 Pro, and Samsung Galaxy S8 (see Table 4). The *Samply Research* app was installed by downloading the app from the respective app stores. Then, the testers joined the “Geofencing test” study and gave the app permission to access the smartphone’s location at any time.

**Table 4:** Smartphone specification

Tester	Phone	Model	Operating system	Version
YS	iPhone	iPhone 11 Pro	iOS	14.3
HH	iPhone	iPhone 12 Pro	iOS	14.3
GO	Xiaomi	Redmi Note 10 Pro	Android	11
NH	Samsung	Galaxy S8	Android	9

##### Survey

The online survey was created in the lab.js experiment builder (https://lab.js.org, Henninger et al., 2021) and hosted on the *Open Lab* platform (https://open-lab.online, Shevchenko, 2022). The survey included one multiple-choice question about the participant’s current location. The possible answers were locations from 1 to 15 (e.g., Location 1, Location 2) and “Other” with an option to specify details. Additionally, we recorded the time and the location coordinates using the mobile browser libraries so that locations were recorded on the same smartphone that was used for testing. We used the mobile browser libraries to access the user’s location, because *Samply* itself does not keep records of absolute geolocation.

#### Analysis plan

The data recorded in *Samply* and the survey were matched using the message ID, a unique identifier for each notification passed via a link and recorded in the survey. We computed the sensitivity score and distance to the center of the geofenced area for each experimental condition. Based on the distance, we later calculated the percentage of the notifications sent at different distances. To test the effect of different conditions, we used the general linear model approach: logistic regression to model the sensitivity as the probability to receive a notification and linear regression to model the distance to the center (R Core Team, 2021). Although the data were nested within evaluators, we did not apply a mixed-effects model approach due to the small number of clusters (*N* = 4). The effect of the environment was of primary interest, given that the absence of Wi-Fi networks in the forest area might impair the geofencing. Turned on Wi-Fi access on the device was expected to have a higher sensitivity than switched off Wi-Fi. We expected a larger distance to the center of the geofenced area for exit events than for enter events. The reason for our expectation was the setup of the experiment. Given a possible time delay between the moment of crossing the fence and notification, people entering the area may be closer to the center of the area than people walking out of the area. We did not expect to see statistically significant differences with regard to the effects of the radius and type of operating system.

### Results

We conducted the tests with the mobile app *Samply Research* between July and November 2021 and obtained 330 records of notifications sent from the server. In 16 of 330 cases, although the notification was delivered, no notification information could be recorded due to the lack of Internet connection, so we treated 16 unidentified records as missing data. Therefore, we used the remaining 314 records to calculate the sensitivity score.

To calculate the distance to the center of the geofenced area, we needed to record the location of the testers. In 46 of 314 cases, it was impossible to record the smartphone location due to an unstable Internet connection^8^. Therefore, we used the other 268 records where the location was recorded to calculate the distance between the center of the geofenced area and the actual position of the smartphone. The sensitivity score and distance for each of the experimental conditions can be found in Appendix Table 16.

#### Sensitivity

On average, the notification was received in 82.50% of all locations^9^. To evaluate the effect of each experimental factor on sensitivity, we constructed a logistic regression model. The dependent variable was binary – whether the notification was received or not. As independent variables in the model, we entered the type of environment (downtown, residential area, and forest), Wi-Fi settings (off, on), type of event (enter, exit), operating system of the smartphone (Android, iOS), and radius (10, 50, and 100 m). The results of the logistic regression model are displayed in Table 5.

**Table 5:** Model 1. Odds ratios for probability to receive a notification

Predictors	Odds ratios	SE	95% CI	*p* value
(Intercept)	0.98	0.41	0.44–2.23	0.96
Forest	0.43	0.40	0.19–0.93	0.035
Residential area	0.71	0.42	0.31–1.60	0.41
Wi-Fi is on	1.17	0.38	0.62–2.21	0.63
Exit event	1.05	0.32	0.56–1.99	0.87
iOS	7.61	0.38	3.76–16.59	< 0.001
50-m radius	7.58	0.40	3.57–17.33	< 0.001
100-m radius	10.94	0.44	4.83–27.73	< 0.001
Observations	360			
*R*^2^ Tjur	0.26			

The odds ratios compare the odds of receiving a notification at the absence and at the presence of the predictor. Odds ratios greater than 1 indicate that receiving a notification is more likely when the predictor is present. Odds ratios less than 1 indicate that receiving a notification is less likely when the predictor is present. *R*^2^ Tjur is a coefficient of discrimination, which represents the difference between the averages of fitted values for successes (notifications received) and failures (notifications not received) (Tjur, 2009)

The baseline condition in the model denotes the enter event in the 10-m area in downtown, on an Android device with turned off Wi-Fi. The sensitivity in the baseline condition was 60%. The effects of other conditions are interpreted in terms of the odds ratio, increasing (odds ratio > 1) or decreasing (odds ratio < 1) the probability to receive the notification in comparison with the baseline condition.

Conducting a study in the forest area decreased the probability to receive the notification, OR = 0.43, 95% CI = 0.19–0.93, *p* = 0.035. Smartphone operating system turned out to play an important role in sensitivity, with iOS devices showing higher sensitivity than Android devices, OR = 7.61, 95% CI = 3.76–16.59, *p* < 0.001***.*** Both 50 and 100 m radius of geofenced area had a significantly higher sensitivity compared to 10-m radius, OR = 7.58, 95% CI = 3.57–17.33, *p* < 0.001, and OR = 10.94, 95% CI = 4.83–27.73, *p* < 0.001, respectively. Neither the Wi-Fi settings nor the type of event significantly affected the probability to receive the notification (*p*s > 0.05).

#### Distance to the center

**Fig. 3:**
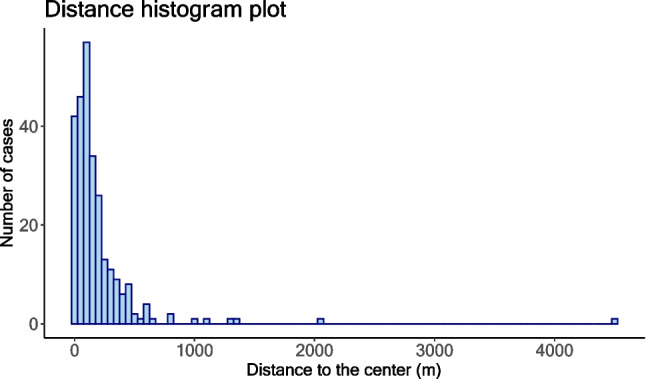
The distribution of distances to the center of the geofenced area

The distribution of distances to the center of the geofenced area was right-skewed due to some potentially invalid data points (see Fig. 3).

For further analysis, we excluded six observations as outliers that were above two standard deviations from the mean (upper threshold of 882.86 m) and could distort the analysis. The average distance in the remaining sample was 153.34 m (Med = 113.08, SD = 146.18). The majority of excluded observations were from the forest area (*n* = 5), so the unusually large distances might be related to the lack of Internet connection and hence a measurement error in the mobile browser in the area. For example, a bad Internet connection^10^ could result in a situation where the notification link was clicked but not completely opened in the mobile browser until the evaluator moved to a place with better Internet access (which may be 1000 m away from the location where the notification was received). In this case, the measurement would not be a valid representation of the distance.

Therefore, we used the remaining 262 records for the analysis in the linear regression model. The dependent variable was the distance between the smartphone’s position and the center of the geofenced area. As independent variables in the model, we entered the type of environment (downtown, residential area, and forest), Wi-Fi settings (off, on), type of event (enter, exit), operating system of the smartphone (Android, iOS), and radius (10, 50, and 100 m). The results of the linear regression model are displayed in Table 6.

**Table 6:** Model 2. Regression coefficients for the distance to the center

Predictors	Estimates	SE	95% CI	*p* value
(Intercept)	28.13	21.95	-15.09 to 71.36	0.20
Forest	75.64	18.86	38.50–112.78	< 0.001
Residential area	27.47	16.10	-4.24 to 59.19	0.089
Wi-Fi is on	-49.72	14.25	-77.79 to -21.66	0.001
Exit event	144.52	14.28	116.39–172.64	< 0.001
iOS	85.48	15.14	55.67–115.30	< 0.001
50-m radius	5.86	18.84	-31.25 to 42.96	0.76
100-m radius	16.71	18.95	-20.61 to 54.02	0.38
Observations	262			
*R*^2^ / *R*^2^ adjusted	0.41/0.39			

It is important to note that the distance data represent only the cases where there was an Internet connection. Because we excluded missing data from locations without an Internet connection, the missing mechanism was not random. Therefore, the results should be considered as the results only for situations with an Internet connection. This limitation generally applies to Internet-based studies, by definition they require an Internet connection.

The baseline condition represents the enter event in the 10-m area in downtown, on an Android device with turned off Wi-Fi. The average distance to the center in the baseline condition was 28.13 m (95% CI = -15.09 to 71.36 m). Being in the forest area increased the distance between the location of receiving a notification and the center of the geofenced area, *b* = 75.64, 95%CI = 38.50–112.78 m, *p* < 0.001. We detected no significant difference between downtown and residential areas, *b* = 27.47, 95%CI = -4.24 to 59.19 m, *p* = 0.089. Turning on Wi-Fi decreased the distance, *b* = -49.72, 95% CI = -77.79 to -21.66 m, *p* = 0.001. With regard to operating system differences, iOS had a larger distance compared to Android, *b* = 85.48, 95% CI = 55.67–115.30 m, *p* < 0.001. Using the 50-m or 100-m radius surprisingly did not affect the distance to the center, *b* = 5.86, 95% CI = -31.25 to 42.96 m, *p* = 0.76, and *b* = 16.71, 95% CI = -20.61 to 54.02 m, *p* = 0.38. Notifications sent on the exit event had a larger distance to the center, *b* = 144.52, 95% CI = 116.39–172.64 m, *p* < 0.001, which was expected as the exit event should be triggered after people left the geofenced area. Please see Appendix Table 17 for the marginal effects of each experimental condition on sensitivity and distance for enter and exit events. We have also calculated the statistical power post hoc using a simulation approach (see Appendix Table 22).

#### False alarms

Regarding false alarms, we did not observe any obvious cases of notification confusion, such as approaching location 1 but receiving a notification for location 2. There was a technical error at the moment of joining the study in the mobile application and enabling notifications for exit events. If the user was outside the area, this resulted in a batch of notifications (15 notifications) being sent to the user at once (as the operating system incorrectly registered the exit event). We discarded these notifications and solved this error afterwards, which will be explained in Study 2.

**Fig. 4:**
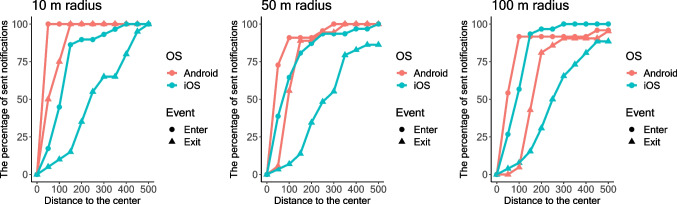
Percentage of sent notifications for different distances to the center of the geofenced area*.* *Note*: On the *X*-axis, the distances between the location where the notification was received and the center of the fenced area are shown. For each distance, we calculated the percentage of notifications (between 0 and 100%, on the *Y*-axis) that had arrived at that or smaller distance

The data on distance to the center of the geofenced area showed that notifications often arrived outside the geofenced area. While this was expected for exit events, notifications for enter events sometimes were also sent outside the area. Therefore, whether a notification is considered a "false alarm" depends on the threshold chosen for when notifications are correctly sent and when they are not. For example, if we apply a strict threshold of the exact geofencing boundary, then only notifications for entering events sent at the boundary or within the geofenced area should be considered correct, and all others are "false alarms". To investigate the relationship between the distance to the center and the number of sent notifications, we calculated the percentage of notifications received at different distances from the center of the geofenced area (see Fig. 4). For example, iOS can potentially generate a higher number of false alarms than Android, which corresponds to the fact that iOS notifications had a larger distance to the center of the geofenced area than Android notifications.

#### Exploratory analysis

**Fig. 5:**
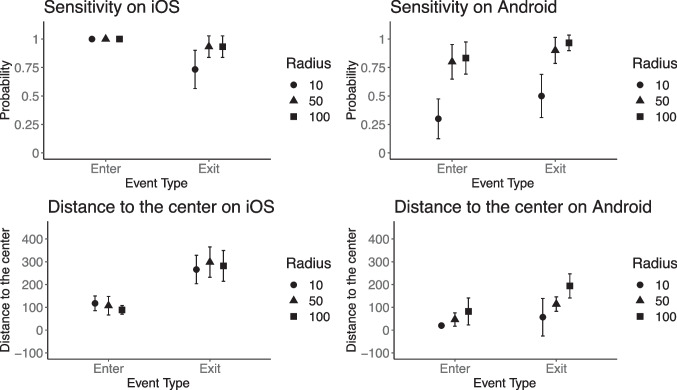
Effect of operating system and radius on sensitivity and distance to the center. *Note*. 95% CI is shown. The probability of receiving a notification indicates the sensitivity of geofencing. The distance to the center of the geofenced area is displayed in meters. The radius in the legend presents the radii of the geofenced area: 10, 50, and 100 m

To investigate further the differences between operating systems, we examined how Android and iOS systems performed with different radii (see Appendix Table 18 for descriptive statistics). We repeated the main analysis with additional interactions between the type of operating system and radius in the subgroups of enter and exit events. Regarding sensitivity, the interactions between the operating system and radius were not statistically significant (*p*s > 0.05). Concerning the distance to the center of the geofenced area, the interaction effect between the type of operating system and 100-m radius was not statistically significant for enter events (*b* = -78.84, SE = 45.49, *p* = 0.09), but reached the level of statistical significance for exit events (*b* = -166.06, SE = 76.22, *p* = 0.032). Android showed higher differentiation between the radii of different sizes, while iOS demonstrated little differentiation between the radii (see Fig. 5). That means, notifications for exit events on Android devices were sent closer to the border of the geofenced area than on iOS devices.

### Discussion

The goal of Study 1 was to test the geofencing method under various conditions, such as the type of environment, geofencing radius, Wi-Fi settings, the type of event, and operating system. Study 1 demonstrated a moderately high geofencing sensitivity, defined as the probability of receiving a notification at a location, of 82.50%. The average distance between the actual location of receiving a notification and the center of the geofenced area was 87 m for enter and 234 m for exit events. Importantly, both sensitivity and the distance were affected by several factors, such as location radius, operating system, environment, Wi-Fi settings, and the type of event.

Using a 10-m radius for geofencing notifications resulted in missed notifications on Android or notifications delivered at a larger distance on iOS (see Table 15 in General discussion for recommendations on using geofencing). In general, iOS outperformed Android in sensitivity, but Android notifications were sent closer to the border. No substantial differences were found between residential and downtown areas, but geofencing in forest areas encountered problems due to a lack of mobile Internet connection. Turned-off Wi-Fi increased the distance for exit events, but did not affect sensitivity. The type of geofencing event (enter or exit) did not influence the probability of receiving the notification, whereas exit events had a larger distance than enter events.

#### Study limitations

Study 1 had some limitations that could have biased the results. First, we used the GPS tracker of the smartphone to measure its absolute geolocation in Study 1. This measure might not be independent, as the same smartphone’s GPS system was used to trigger notifications. In Study 2, therefore we used an independent external GPS tracker to record the position of the evaluator.

Additionally, the distance between the location of receiving a notification and the center of the geofenced area may not be an ideal measure of geofencing precision. If the notification is triggered by the event of crossing the geofencing border when entering or leaving the area, a more precise measure would be the distance between the location of the fence crossing and the location where a notification was received. In Study 1, we could not calculate that distance because we measured the actual location of the smartphone only at the moment of opening a web survey after receiving the notification. In Study 2, we thus recorded the evaluator’s complete path with an external GPS tracker. This allowed us to calculate where and when the geofenced area was crossed. Because of this, we could further calculate the distance and time difference between the location and time of crossing the fence and the location and time of receiving the notification.

In Study 1, the testers moved through a geofenced area without stopping or taking a break. The testers were free to choose their own speed, there were no specific instructions regarding the time or speed. However, in a real case scenario people might stay for some time inside of a geofenced area (e.g., visiting a shop, staying in the office). Therefore, we added a staying inside condition in Study 2. In this condition, we investigated a more realistic protocol that represented the situation in which people are not just going through the geofenced locations, but stay inside of them for some time^11^. From the perspective of geofencing technology, it might make a difference, as staying inside of a geofenced area for a longer time can increase the likelihood that an enter event will be triggered.

## Study 2

### Method

#### Participants

All the tests were performed by the first author together with a research assistant to ensure following the same protocol.

#### Procedure

The procedure was identical to Study 1, except we introduced some changes to improve the methodology. First, an external GPS tracker was used to record evaluators’ timestamps and positions during the whole test at the rate of one recording per second. Second, we added a new condition, where evaluators stayed for 5 min in the center of each location.

As Study 1 had clearly demonstrated the disadvantage of geofencing in the forest environment, where there was a lack of Internet, we excluded the forest from the tests. Additionally, as Study 1 did not find any differences between downtown and residential areas, we conducted all tests in the downtown area. We also left out the condition with turned off Wi-Fi settings, as there were no substantial differences found for each condition in Study 1, and participants usually have their Wi-Fi turned on.

We systematically varied the factors *type of geofencing event* (enter or exit), *type of OS* (Android, iOS), and *user behavior* (walking through or staying in the center for 5 min). Therefore, the route in the downtown environment was completed eight times in a fully crossed design with two event types, two operating systems, and two user behavior conditions (2 x 2 x 2). To evaluate the effect of the geofence radius, there were 15 locations for each route – five locations for each radius of 10, 50, and 100 m (see Fig. 2A). In total, 120 tests were performed (1 environment x 8 repetitions x 15 locations).

During the tests, both evaluators walked along the pathway in the downtown area that went through predefined geofenced areas (see Fig. 2A). The smartphone was carried in the right hand, and the evaluator was not to use it for other purposes (e.g., texting or web browsing). The Garmin Watch (model Venu Sq), an external GPS tracker, was carried on the left hand. When the evaluator heard the signal of notification arrived or/and felt vibration of the smartphone, he stopped moving, and registered the event on the Garmin Watch by starting a new lap. Then the evaluator noted the time of receiving the notification and the lap number in the Garmin Watch on a protocol sheet. The notification was then removed from the screen by swiping it away.

We measured the geofencing sensitivity by recording whether the notification was sent for every location the evaluator visited. We also computed the distance and time difference between the actual location of the evaluator at the moment of receiving the notification and the point of crossing the area fence. False-alarm rates were later calculated based on the distance data.

#### Materials

##### Smartphones

The evaluators used their own smartphones: iPhone 11 and Nokia (see Table 7).

**Table 7:** Smartphone specification in Study 2

Evaluator	Phone	Model	Operating system	Version
HH	iPhone	iPhone 11 Pro	iOS	14.3
YS	Nokia	Nokia 7.1 (TA-1095)	Android	9

##### External GPS tracker

We used a Garmin Watch (model Venu Sq) as an external GPS tracker. The log files recorded by the watch were available for downloading in the Garmin Connect software. The log files were opened and examined with the Google Earth program. Each event of a notification was recorded as the start of a new lap, which was shown in the logs with a timestamp and geolocation. The time and location of crossing the virtual fence were also calculated from the logs.

#### Preventing false alarms for exit events

To prevent the batch notification problem observed in Study 1, we implemented the “vicinity zone” algorithm in the *Samply Research* mobile app. In general, for each triggered notification in the mobile app, the operating system provides additional information about whether the user is inside or outside the geofenced area. While this prevents a false alarm for enter events (by not sending a notification if the user is outside), for exit events the condition that the user is outside is quite loose and can be met if the user is, for example, 100 m or 100 km away. As a remedy and to provide an additional control mechanism, we programmed the app to check the user’s current location when an exit event is triggered. Only when the user is in the immediate vicinity of the fence (i.e., up to 200 m) can the application proceed and send a notification. The exact threshold of 200 m was based on the results of Study 1 in the areas with good Internet connectivity (downtown and residential area). The idea behind this is that if the user has actually left the area, the user should still be somewhere nearby. If this is not the case, a false alarm is very likely, and the notification should not be sent.

#### Analysis plan

The data recorded in *Samply* and the Garmin Watch were matched using the time and the lap numbers recorded during the tests. We computed sensitivity, distance, and time difference for each radius condition. The distance and time difference to the fence were calculated with respect to the location and time at which the fence was crossed. Positive values on the distance and time scales mean that the notification was triggered after the participants had crossed the fence and negative values mean that the notification was triggered before they had crossed the fence. Analysis was similar to the analysis in Study 1. In general, we expected to replicate the results of Study 1. With respect to the new staying inside condition, we expected to record higher sensitivity.

### Results

We conducted the tests in July and August 2022 and obtained 84 records of notifications sent from the server. One notification was sent twice, we only used the first notification in the analysis. The final dataset thus consisted of 83 records. For each of the notifications, we recorded the absolute position of the smartphone, and calculated the distance and time difference with the location where the fence was crossed. The sensitivity score, distance, and time difference for each of the experimental conditions can be found in Appendix Table 19.

#### Sensitivity

On average, the notification was received in 69% of all locations. To evaluate the effect of each experimental factor on sensitivity, we constructed a logistic regression model. The dependent variable was binary – whether the notification was received or not. As independent variables in the model, we entered the type of user behavior (no waiting, 5 min waiting), the type of event (enter, exit), operating system of the smartphone (Android, iOS), and radius (10, 50, and 100 m). The results of the logistic regression model are displayed in Table 8.

**Table 8:** Model 1. Odds ratios for probability to receive a notification

Predictors	Odds ratios	SE	95% CI	*p* value
(Intercept)	0.18	0.12	0.05–0.61	0.009
Waiting 5 min	9.23	5.51	3.08–32.78	< 0.001
iOS	18.15	11.64	5.69–72.43	< 0.001
Exit event	0.18	0.10	0.05–0.51	0.002
50-m radius	4.26	2.71	1.28–15.88	0.023
100-m radius	13.00	9.52	3.39–61.95	< 0.001
Observations	120			
*R*^2^ Tjur	0.47			

The odds ratios compare the odds of receiving a notification at the absence versus the presence of the predictor. Odds ratios greater than 1 indicate that receiving a notification is more likely when the predictor is present. Odds ratios less than 1 indicate that receiving a notification is less likely when the predictor is present. *R*^2^ Tjur is a coefficient of discrimination, which represents the difference between the averages of fitted values for successes (notifications received) and failures (notifications not received) (Tjur, 2009)

Waiting 5 min in the center of the geofenced location increased the probability to receive the notification, OR = 9.23, 95% CI = 3.08–32.78, *p* < 0.001. The iOS device received more notifications than the Android device, OR = 18.15, 95% CI = 5.69–72.43, *p* < 0.001. Notifications for exit events arrived less often than for enter events, OR = 0.18, 95% CI = 0.05–0.51, *p* = 0.002. Finally, both 50-m and 100-m radius of geofenced area triggered more notifications than the 10 m radius, OR = 4.26, 95% CI = 1.28–15.88, *p* = 0.023, and OR = 13.00, 95% CI = 3.39–61.95, *p* < 0.001, respectively.

#### Distance to the fence

The average distance between the location where the fence was crossed and the location where the notification was received was -6.76 m for enter events (range -137.79 to 109.47, Med = 8.76, SD = 71.13) and 132.00 m for exit events (range -56.58 to 217.56, Med = 140.75, SD = 53.99). In the regression model analysis, the dependent variable was the distance between the smartphone’s position and the fence. As independent variables in the model, we entered the type of user behavior (no waiting, waiting 5 min), the type of event (enter, exit), operating system of the smartphone (Android, iOS), and radius (10, 50, and 100 m). The results of the linear regression model are displayed in Table 9.

**Table 9:** Model 2. Regression coefficients for the distance to the fence

Predictors	Estimates	SE	95% CI	*p* value
(Intercept)	-8.97	20.21	-49.21 to 31.27	0.66
Waiting 5 min	0.32	13.77	-27.10 to 27.75	0.98
iOS	-41.80	14.20	-70.06 to -13.53	0.004
Exit event	135.40	13.32	108.88–161.93	< 0.001
50-m radius	41.12	17.30	6.66–75.57	0.02
100-m radius	38.15	17.25	3.80–72.50	0.03
Observations	83			
*R*^2^/*R*^2^ adjusted	0.63/0.60			

The baseline condition represents the enter event without waiting in the 10-m area, on an Android device. The average distance to the fence in the baseline condition was -8.97 m, so the notification was on average triggered before crossing the fence (95% CI = -49.21 to 31.27 m). Waiting 5 min in the center of the geofenced area did not affect the distance, *b* = 0.32, 95% CI = 27.10 to 27.75 m, *p* = 0.98. iOS triggered notifications at a greater distance before crossing the fence compared to the baseline with an Android device, *b* = -41.80, 95% CI = -70.06 to -13.53 m, *p* = 0.004. Notifications sent on the exit event had a larger distance to the fence after crossing than notifications sent on the enter event, *b* = 135.40, 95% CI = 108.88–161.93 m, *p* < 0.001. The 50-m or 100-m radius increased the distance after crossing the fence compared to the 10-m radius in the baseline condition, *b* = 41.12, 95% CI = 6.66–75.57 m, *p* = 0.02, and *b* = 38.15, 95% CI = 3.80–72.50 m, *p* = 0.03.

#### Time difference with the fence crossing

For the analysis, we had to exclude two observations, because of unusually large time difference values, which were not related to the experiment (i.e., taking a break during the test). The average time difference between the time when the fence was crossed and the time when the notification was received was 21.68 s for enter events (range -172 to 684, Med = 20.0, SD = 147.88) and 177.82 s for exit events (range -59 to 560, Med = 176.50, SD = 109.85). In general, the linear regression analysis of time differences confirmed some of the patterns that we had found in the analysis of the distance to the fence above. The average time difference with the fence in the baseline condition was 46.09 s, so the notification was on average triggered after crossing the fence (95% CI = -33.61 to 125.79 s). iOS had triggered notifications before crossing a fence compared to Android, *b* = -122.35, 95% CI = -178.81 to -65.89 s, *p* < 0.001 (see Table 10). Notifications sent on the exit event had a larger time difference after crossing the fence than notifications sent on the enter event, *b* = 160.49, 95% CI = 107.45–213.52 s, *p* < 0.001. There were no differences in the time between 50- or 100-m radius compared to the 10-m radius (although it took longer for both radii). Waiting 5 min in the center of the geofenced area did not affect the time difference as well, in comparison with the condition of going through the geofenced area without stopping. Please see Appendix Table 20 for the marginal effects of the conditions and Appendix Table 22 for statistical power estimates.

**Table 10:** Model 3. Regression coefficients for the time difference with the fence crossing

Predictors	Estimates	SE	95% CI	*p* value
(Intercept)	46.09	40.01	-33.61 to 125.79	0.25
Waiting 5 min	31.88	27.27	-22.44 to 86.19	0.25
iOS	– 122.35	28.34	-178.81 to -65.89	< 0.001
Exit event	160.49	26.62	107.45–213.52	< 0.001
50-m radius	62.33	34.36	-6.11 to 130.78	0.07
100-m radius	28.18	33.94	-39.44 to 95.80	0.41
Observations	81			
*R*^2^/*R*^2^ adjusted	0.46/0.42			

Estimates are given in seconds

#### False alarms

**Fig. 6:**
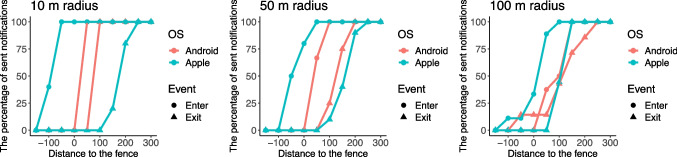
Percentage of received notifications for different distances to the fence*.* *Note*. On the *X*-axis, the distances between the location where the notification was received and the fence are shown. For each distance, we calculated the percentage of notifications (between 0 and 100%, on the *Y*-axis) that had arrived at that or smaller distance

Regarding false alarms, one notification was sent twice with an interval of 1 min 33 s. It was triggered by an exit event on Android device at the location of 100-m radius in the condition when the tester moved through the location without stopping in the center. As in Study 1, the data on the distance to the fence showed that notifications often arrived before the smartphone crossed the fence. Whether to consider these notifications as false alarms depends on the threshold a researcher selects. To investigate the relationship between the distance to the fence and the false alarm rate, we calculated the percentage of notifications received at each distance (see Fig. 6).

#### Exploratory analysis

**Fig. 7:**
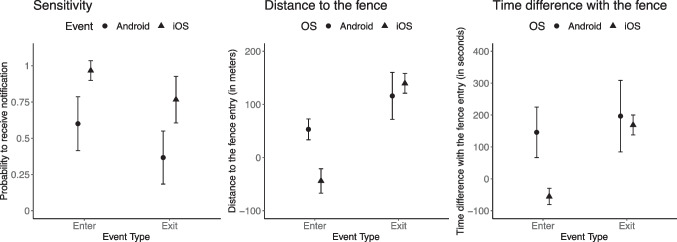
Sensitivity, distance, and time difference for iOS and android operating systems for enter and exit events. *Note*. 95% CI is shown

When comparing distances and time differences for enter events, the average distance was negative, but the average time difference was positive. This counterintuitive outcome could be due to averaging across different operating systems. A clearer picture arises if we consider the effects separately for iOS and Android operating systems (see Fig. 7).

We repeated the main analysis with additional interactions between the operating system and event. The interaction was statistically significant both for the distance, *b* = 120.76, SE = 23.98, *p* < 0.001, and for the time difference, *b* = 168.72, SE = 52.12, *p* = 0.002. With regard to enter events, iOS sent notifications on average before the fence was crossed, as shown by the negative distance to the fence (M_iOS_ = -43.90, SD_iOS_ = 60.25) and time difference (M_iOS_ = -55.34, SD_iOS_ = 66.93). Android sent notifications on average after the fence was crossed, as indicated by the positive distance (M_Android_ = 53.07, SD_Android_ = 39.57) and time difference (M_Android_ = 145.78, SD_Android_ = 159.27).

### Discussion

The goal of Study 2 was to confirm the findings of Study 1 using an external GPS tracker, and in addition systematically test the difference between walking through the geofenced area or staying for 5 min in the center of the location. Study 2 replicated the differences in sensitivity between iOS and Android operating systems, with iOS having a higher sensitivity than Android. A larger radius and waiting 5 min increased sensitivity, while exit events generally had a lower sensitivity level. Study 2 also provided more accurate measurements of distance and confirmed that iOS triggered enter notifications before crossing the fence, whereas Android sent enter notifications after crossing the fence. Possible explanations and recommendations are discussed in the General discussion section.

## Study 3

In Studies 1 and 2, we only tested a limited selection of smartphones with trained testers who paid extensive attention to receiving notifications. However, in many studies researchers might encounter a higher variation of participant devices, operation system versions, and mobile Internet providers. The question is whether and to which extent geofencing will still be accurate under those conditions. To evaluate the geofencing method, we conducted a study with participants, who were naïve to the research materials and hypotheses. The participants, students of the University of Konstanz, used their own smartphones.

### Method

#### Participants

Fifty-eight participants (54 women, four men, mean age 21, SD = 4, range 18–42) completed the baseline survey and registered in the mobile app “Samply Research”. Regarding the type of operating system, 40 participants used iOS, 17 participants used Android, and one participant used EMUI (an Android-based system developed by Huawei). From the perspective of the system sending notifications, at least one geofencing notification was sent to 53 participants, and the average number of notifications sent per participant was 15.83, Med = 14, SD = 13.05, range 1–71. As for responding to the notification by entering the data in the geofencing survey, 49 participants responded at least once, and the average number of completed geofencing surveys per participant was 12.31, Med = 12, SD = 7.25, range 1–39.

#### Procedure

After completing the baseline survey, participants installed the *Samply Research* mobile application and joined the study in the app. From that point on, participants received a notification linked to the geofencing survey when they entered or left the area of interest, i.e., the University of Konstanz. The radius was set up to 200 m. In addition, participants received a notification at 9:00 pm each evening with a link to the daily survey. The study lasted 2 weeks for each participant, and participants could begin the study between November 28 and December 7, 2022. At the end of the study (on day 15) participants received a notification with a link to the debriefing survey.

#### Materials

All surveys were programmed in the lab.js experiment builder (https://lab.js.org, Henninger et al., 2021) and hosted on the *Open Lab* platform (https://open-lab.online, Shevchenko, 2022).

##### Baseline survey

The purpose of the baseline survey was to introduce participants to the study and to collect basic socio-demographic (gender, age) and smartphone-related technical information (smartphone model, operating system, mobile Internet provider). The perceived quality of mobile Internet connectivity was measured on a five-point Likert scale ranging from “very poor” to “excellent”. The survey also explained the goal and content of the study to participants, provided the informed consent form, and instructed them on how to install and use the mobile application.

##### Geofencing survey

The survey consisted of two questions about the participant’s location when the notification was received. The first question asked whether the participant was at the university or not. The second question was whether the participant was entering, leaving, or neither entering nor leaving the university.

##### Daily survey

The daily survey aimed to ask participants whether they were at the university during the day. The options were “Yes”, “No”, and “Don’t want to answer”. The notification to fill in the daily survey was sent out at 9:00 pm on each study day.

##### Debriefing survey

In the debriefing survey, participants were informed about the study’s aim and thanked for their contribution. Participants were asked whether there were any technical problems during the study and whether they had any comments about the study for researchers. The survey also included instructions on how to disable geofencing, leave the study, and delete the app.

#### Analysis plan

The data recorded in *Samply* and online surveys were matched using the anonymous participant ID. We computed the confusion matrix for enter and exit events based on notification logs and participants’ survey answers. Hits and false alarms were calculated from the answers that participants provided in the geofencing surveys, i.e., whether they were entering/leaving the university at the moment of receiving the notification. Misses and correct rejections were computed from the answers that participants provided in the daily surveys, i.e., whether they were at the university during the day. Sensitivity and precision statistics were calculated for each participant (see Appendix Table 21) and for each phone model. We used a *t* test to analyze the effect of the type of operating system on the sensitivity and precision of enter and exit events.

### Results

#### Sensitivity

To compute sensitivity, we calculated the number of correct hits and misses based on the answers to the daily survey (see Table 11). For enter events, the sensitivity was 70%, and for exit events, the sensitivity was 18%.

**Table 11:** Geofencing notifications and participants’ responses to the daily surveys

	Participant’s response		
	Was at the university during the day	Was not at the university during the day	Total
Enter events			
At least one enter notification was sent during the day	215 (40.0%)	4 (0.7%)	219 (40.7%)
No enter notification was sent during the day	93 (17.3%)	226 (42.0%)	319 (59.3%)
Total	308 (57.3%)	230 (42.7%)	538 (100%)
Exit events			
At least one exit notification was sent during the day	54 (10.0%)	0 (0%)	54 (10%)
No exit notification was sent during the day	254 (47.2%)	230 (42.8%)	484 (90%)
Total	308 (57.2%)	230 (42.8%)	538 (100%)

#### Precision

To compute precision, we calculated the number of correct hits and false alarms based on the answers to the geofencing survey (see Table 12). The precision for enter events was 70% and for exit events 89%.

**Table 12:** Geofencing notifications and participants’ responses about their current movement in the geofencing surveys

	Participant’s response				
	Entering the university	Leaving the university	Neither entering nor leaving	Do not know	Total
An enter notification was sent	272 (56.9%)	23 (4.8%)	81 (16.9%)	13 (2.7%)	389 (81.3%)
An exit notification was sent	6 (1.3%)	79 (16.5%)	3 (0.7%)	1 (0.2%)	89 (18.7%)
Total	278 (58.2%)	102 (21.3%)	84 (17.6%)	14 (2.9%)	478 (100%)

It could be that some participants received multiple enter notifications even though they had been at the university for some time, so they did not select the “Entering the university” option. Analysis of responses to the second question in the geofencing survey about the current position confirmed that participants were at the university 97% of the time when enter notifications were sent (see Table 13). Thus, the false alarms for enter events were not related to misidentification of location (as participants were at the university), but were related to either timing (e.g., a notification was delayed) or the repetition of a notification when participants had been in the university for some time or had moved on campus. For exit events, 91% of participants reported being at the university at the time they received the notification. The boundaries between the university and outside the university were not clearly defined in the study, so participants who were leaving the university could still have considered that they were in the university campus area.

**Table 13:** Geofencing notifications and participants’ responses about their current location in the geofencing surveys

	Participant’s response			
	At the university	Not at the university	Do not know	Total
An enter notification was sent	379	9	1	389
	(79.2%)	(1.9%)	(0.2%)	(81.3%)
An exit notification was sent	81	7	1	89
	(17.0%)	(1.5%)	(0.2%)	(18.7%)
Total	460	16	2	478
	(96.2%)	(3.4%)	(0.4%)	(100%)

#### Smartphone models

**Fig. 8:**
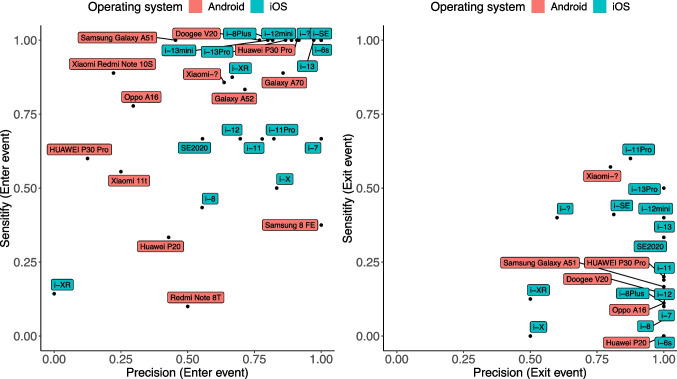
Precision and sensitivity for enter and exit events*. Note.* The smartphone model names are shown next to the data points. iPhone models are abbreviated with “i-”. Question marks in the name of a phone model (e.g., “Xiaomi-?”) mean that the participant has not provided information about the specific model of the phone

To explore the variability in precision and sensitivity between smartphones, we calculated both scores for each smartphone model for enter and exit events (see Fig. 8).

#### Operating systems

We conducted *t* tests to analyze whether there were differences in precision and sensitivity between the iOS and Android operating systems. The precision for enter events and the sensitivity for exit events were higher for iOS devices than for Android devices, *t* (*df* = 21.11) = – 2.35, *p* = 0.028, and *t* (*df* = 40.90) = – 2.57, *p* = 0.014 (see Table 14 and Appendix Table 22 for statistical power estimates). Precision for exit events and sensitivity for enter events did not differ statistically significantly between operating systems,* t* (*df* = 17.07) = 1.00, *p* = 0.33, and *t* (*df* = 25.11) = – 1.27, *p* = 0.21. The perceived quality of the mobile Internet connection or the type of mobile Internet provider had no effect on precision and sensitivity (*p*s > 0.05).

**Table 14:** Sensitivity and precision for iOS and android operating systems

Event	Measure	Android, M (SD)	iOS, M (SD)
Enter	Precision	0.55 (0.29)	0.77 (0.28)
Enter	Sensitivity	0.58 (0.39)	0.72 (0.34)
Exit	Precision	0.97 (0.08)	0.92 (0.17)
Exit	Sensitivity	0.07 (0.15)	0.21 (0.22)

### Discussion

The goal of Study 3 was to investigate the feasibility of the geofencing method, which was implemented via *Samply*, in a study with naïve subjects. At the same time, because the participants used their own smartphones, we tested the app’s functionality on different devices, operating systems, and mobile Internet providers. The general results indicate a moderate level of sensitivity and precision for enter events, 70% for both. For exit events, while precision was high at 89%, sensitivity was low at 18%. As in Studies 1 and 2, iOS performed better than Android. The iOS operating system demonstrated higher precision for enter events and higher sensitivity for exit events than Android.

## General discussion

Our three empirical studies showed that implementing geofencing technology yielded sensitivity and precision scores comparable to those of previous studies (Nguyen et al., 2017; Suyama & Inoue, 2016; Wray et al., 2019). The scores were not ideal, suggesting that there is room for improvement in both the technology and its use by researchers. We identified several limitations and provided recommendations for geofencing research (see Table 15).

**Table 15:** Limitations and recommendations for geofencing research

Factor	Limitations	Recommendations
Radius	A 10-m radius can be problematic, leading to missed notifications on Android and false alarms on iOS	Use a radius of 100 m or higher
Operating system	Some Android devices may interfere with sending notifications, which can reduce sensitivity	Record the operating system and device type. Prepare troubleshooting instructions for participants. Pre-register operating systems and device types that are accepted in providing data for a study
Environment	Missing Internet connection, network congestion, or blocked GPS signal may delay notifications	Avoid areas without a mobile Internet connection or with network congestion. Set up the geofencing border in an open area
Wi-Fi settings	Turned off Wi-Fi may delay notifications for exit events	Ask participants to keep their Wi-Fi on
The type of event (enter, exit)	Exit notifications have a longer delay than enter notifications	Take into account that exit notifications may result in a higher variability of responses than enter notifications
User behavior	Passing through the location may decrease sensitivity, specifically for Android	Apply geofencing to areas where participants would stay for at least 5 min
General	False alarms	Avoid overlapping of geofencing areas. Use a minimum time window in Samply, e.g., 8 h. Customize the vicinity zone for exit notifications in Samply, e.g., 500 m. Ask control questions, e.g., "Are you at the location now?"
	Misses	Ask control questions in a survey at the end of the day, e.g., "Have you been at the location during the day?"
	Potential violation of participant privacy	Provide a study consent form
	Operating systems limit the number of locations	Use fewer than 20 locations per participant/device

### Location radius

Using a 10-m radius for sending notifications may be problematic, as some of the notifications for this radius were not delivered on Android or were delivered earlier than expected on iOS. A 50-m radius improves sensitivity on Android, but iOS still sends notifications earlier than expected. The differences between operating systems can be attributed to the operating systems’ underlying strategies for optimized geofencing. As Android requests the current location every second minute, locations with a 10-m radius could be missed because the smartphone had a higher chance of being removed from the zone before the notification is triggered. Regarding the iOS performance, Apple documentation points out that certain conditions should be met to deliver a notification: “The user’s location must cross the region boundary, move away from the boundary by a minimal distance, and remain at that minimum distance for at least 20 s”^12^. However, the documentation does not explain why notifications are delivered before crossing the region boundary. It might be that iOS uses a radius of 100 m for all radii below 100 m. This would explain the results of Studies 1 and 2, where iOS did not differentiate between radii below 100 m. If this is true, then iOS can reach a higher sensitivity even for smaller radii, such as a 10-m radius, by giving up on precision.

This implies that researchers who want to use geofencing for very specific and small locations, e.g., a smoking area next to an office building, should be aware of the possibility of misses (in particular, for Android smartphones) or false alarms (in particular, for iOS smartphones). Both sensitivity and distance measures were acceptable for a radius of 100 m or above, which leads us to recommend 100 m as a minimum radius for studies. We expect a further increase in radius should not significantly affect the results, although this has to be tested in further research. Many locations can be set up with a radius of 100 m and higher, such as public areas, offices, apartments, stores, etc. Whether the precision can be improved in the future is an open question, as it is not only a matter of technical affordances, but also an ethical decision regarding user privacy.

### Operating system

All three studies reported here demonstrated that iOS currently has an advantage over the Android system in using geofencing for research. These differences between operating systems were mainly present for a 10-m radius in the controlled tests and were also replicated in a study with naïve participants. As discussed above, the differences can be explained by the fact that iOS relies more on distance measures to optimize the geofencing, whereas Android reduces the frequency of tracking (to every second minute or every sixth minute, if the device has not been active) and may apply additional optimization techniques that interfere with geofencing. Android’s doze mode might contribute to misses, although the documentation specifies that the system exits the doze mode when the user moves the device^13^. Moreover, some Android devices optimize battery performance by shutting down or delaying background tasks, such as geofencing. In these cases, researchers may prepare troubleshooting instructions for participants to adjust the settings^14^.

### Environment

We did not find any substantial differences in geofencing sensitivity and distance between residential and downtown areas. Although there were more public Wi-Fi spots in downtown due to stores and restaurants, this did not significantly improve sensitivity and the distance compared to the residential area with fewer public Wi-Fi spots. On the other hand, we discovered that geofencing in the forest area might encounter a number of problems. First, the sensitivity was lower, and the distance was measured as larger in the forest area. Second, in our tests in the forest area, there was also an area without cellular Internet connection, which further reduced connectivity. While the notifications arrived even in the area without Internet connection, the lack of connection prevented the data from being sent from the smartphone to the server and the geolocation from being recorded correctly. Therefore, it is recommended that studies be conducted in an area that has a cellular Internet connection (at least at the poor signal level indicated by at least one bar or dot of signal strength in the smartphone menu).

Because Internet connectivity can affect geofencing technology, it is important to understand what can affect the connection. The signal can be blocked by building materials such as steel, concrete, brick, wood, or fiberglass insulation. Therefore, we recommend setting up a geofencing boundary in an open area. For example, setting up the boundary inside a building would not be a good idea. If researchers are interested in a particular building (e.g., a school, a hospital, a store), the boundary should be set up around the building, including the open space next to the building. In addition, in densely populated areas, cell towers may reach their capacity limit. When many people use the bandwidth of the same network and cell tower, connectivity decreases. This means that geofencing studies could be problematic at events or high-traffic locations (e.g., concerts, conferences, shopping malls) unless Wi-Fi networks compensate for cellular network congestion.

### Wi-Fi settings

Although the previous studies and operating system documentation pointed to possible adverse effects of turned-off Wi-Fi signals, we could only confirm the negative impact of turned-off Wi-Fi on the distance to the center of the geofenced area in exit events. This factor may not be as problematic for geofencing studies because most people usually leave their Wi-Fi on by default. Researchers may remind participants to keep their Wi-Fi on during the study.

### Enter vs. exit event

While the type of geofencing event (enter or exit) did not influence the probability of receiving the notification, it did affect where the geofencing event was triggered. Exit events had a larger distance from the center of the geofenced location than enter events. Given the time delay by the operating system, people leaving the area moved further away from the border with each additional minute. This means that exit notifications can generate a higher variability of responses than enter notifications. When an exit notification arrives, people may be in different locations and at different distances from the geofenced area depending on the mode of transportation (e.g., walking or driving).

By combining enter and exit events, researchers can design studies that measure the time participants spend at a location. To record events without sending notifications, researchers can activate the *invisible mode* for each type of event in *Samply*.

### User behavior

Waiting 5 min in the center of the geofenced area improved sensitivity because smartphones had more time to detect entering or leaving the area. This means that researchers using geofencing should consider how much time participants might spend at the location: The more time a participant spends at the location, the higher the sensitivity likely will be. If a participant only passes through the location, iOS devices will tend to have a higher sensitivity than Android devices.

### General recommendations

There are two general strategies for conducting geofencing research. The researcher can use prepared devices (e.g., smartphones) or utilize participants’ smartphones. In the case of the first strategy, the choice of operating system and smartphone model is important. Our results show that iOS has an advantage over the Android system but comes with higher costs. An Android device could also be an option, as long as a minimum 50-m radius is used and the smartphone is configured to allow geofencing background tasks to run without being optimized by the operating system. With a predetermined smartphone model, researchers can pre-test the phone’s functionality. If researchers choose to use participants’ smartphones, it is recommended to record the type of device and assist participants in adjusting smartphone settings if needed.

False alarms and misses are currently unavoidable in geofencing research. To control for false alarms, researchers can include a question in a geofencing survey that confirms that the participant is in the intended location. To control for misses, researchers can use *Samply’s interval-based notifications functionality* (as we did in Study 3) to ask participants to report whether they were at the intended location during the day. In *Samply*, we have implemented two customizable features to decrease the number of false alarms. These are the *minimum time window* between notifications and the *size of the vicinity zone for exit notifications*. The first feature allows researchers to set a minimum time window between geofencing notifications. When the notification is triggered, an algorithm checks the time of the previous notification and prevents sending the notification if the time that has passed since is too short. The second feature allows users to customize the size of the vicinity zone for exit notifications. Only when a participant is in the vicinity zone (i.e., up to 500 m) could the app proceed and send an exit notification. This enables researchers to control the trade-off between precision and sensitivity for exit events.

Researchers can set up either shared or participant-specific locations. In Study 3, we tested a single location shared among all participants. For those interested in participant-specific locations, the *Samply* mobile app interface allows participants to enter their locations in the app. The location coordinates are not shared with the researcher. This allows for various research designs, such as notifying participants when they enter their office, place of study, home, etc. However, if the geofenced areas are close to each other, the probability of false alarms is higher. Due to the limited number of locations a user can create (20 locations for iOS, 100 locations for Android), we recommend focusing on the most important locations.

Participant privacy is an essential aspect of a geofencing study. Researchers should be explicit and specific about any information they collect in the study, as required by the EU GDPR (Voigt & dem Bussche, 2017). To support this, the *Samply* user interface prompts researchers to provide a study consent form and a rationale for geofencing as two separate information inputs to make it explicit for participants.

### Validation method

To validate the accuracy of geofencing in *Samply*, we combined controlled repeated tests from Study 1 and Study 2 with real-world tests in Study 3. Controlled tests allowed us to systematically examine the combination of different factors, such as radius or operating system. We recommend applying an external source of validation, such as an external GPS tracker in Study 2, to verify an app’s functionality. Although the smartphone can provide various measurements, such as GPS position from the mobile browser, an external data source guarantees the independence of those measurements.

With real-world tests, on the other hand, we estimated the plausibility of applying geofencing given the heterogeneity of devices and user behavior. This approach of combining controlled and real-world tests has been used before with other smartphone apps (e.g., Geyer et al., 2022). However, in our studies, we found that the statistical power of real-world tests in Study 3 was lower than that of controlled tests in other studies (see Appendix Table 22). This difference may be due to the use of aggregated proportions as measures of sensitivity and precision for each participant. While aggregated per-participant data is a common way to measure accuracy, further studies may consider recruiting a larger sample size to increase the statistical power.

## Limitations and further research

The required Internet connectivity is a general limitation of the geofencing approach implemented in Samply. However, given technological developments and the widespread use of smartphones, it is reasonable to assume that more people will own smartphones and have access to mobile Internet. At the same time, we expect the geofencing technology to evolve both quantitatively (by improving sensitivity and precision) and qualitatively, by developing new forms of geofencing (e.g., vertical geofencing in Stieger & Reips, 2019) or combining it with other device information such as spatial orientation (Kuhlmann et al., 2020).

Given our findings and the speed of technological development, further research will be required to amend our findings and test new technological solutions and implementations. At the same time, we are confident that as technology advances, researchers will adopt geofencing (e.g., by using the *Samply* software) to develop and test new questions that complement laboratory research with inquiries into everyday life.

## Conclusion

Technically, geofencing and geotracking technology have already achieved impressive results and are being used in many domains outside of research, e.g., logistics, retail, car navigation, and business (e.g., Uber, Airbnb). Behavioral science research has yet to take advantage of this rapidly evolving technology to explore new applications. User privacy is of great importance, so a technology such as geofencing could be more attractive to users because it does not record absolute geolocation and does not share the data with others. However, although geofencing has this advantage, it needs to be explained to the end user to establish a trustworthy and transparent relationship between the researcher and the participant. People differ in terms of their preferences regarding the level of privacy (Joinson et al., 2006; Paine et al., 2007), so all participants’ privacy should be treated with care.

This manuscript shows advantages, conditions, and limitations of geofencing methodology. As an application ready to be used, *Samply* provides a research infrastructure for geofencing, and researchers are welcome to use the software, run benchmark tests, and use the tool in their studies. Documentation with information on geofencing and step-by-step tutorials can be found on the *Samply* website https://samply.uni-konstanz.de/.

## Data Availability

None of the experiments was preregistered.

## Appendix

**Table 16:** Sensitivity and distance to the center in Study 1

	Environment	Wi-Fi	Event	OS	Radius (m)	Sensitivity (mean)	Sensitivity (SD)	Distance (mean)	Distance (SD)
1	Downtown	On	Enter	Android	10	0.80	0.45	22.46	7.45
2	Downtown	On	Enter	Android	50	0.80	0.45	34.30	33.39
3	Downtown	On	Enter	Android	100	1.00	0.00	28.76	25.23
4	Downtown	On	Enter	iOS	10	1.00	0.00	119.13	85.88
5	Downtown	On	Enter	iOS	50	1.00	0.00	115.27	145.80
6	Downtown	On	Enter	iOS	100	1.00	0.00	85.25	36.14
7	Downtown	On	Exit	Android	10	0.00	0.00	NA	NA
8	Downtown	On	Exit	Android	50	1.00	0.00	82.88	24.80
9	Downtown	On	Exit	Android	100	1.00	0.00	145.10	18.08
10	Downtown	On	Exit	iOS	10	1.00	0.00	127.03	86.24
11	Downtown	On	Exit	iOS	50	1.00	0.00	213.92	54.41
12	Downtown	On	Exit	iOS	100	1.00	0.00	183.15	33.17
13	Downtown	Off	Enter	Android	10	0.60	0.55	20.04	11.14
14	Downtown	Off	Enter	Android	50	1.00	0.00	28.26	17.83
15	Downtown	Off	Enter	Android	100	1.00	0.00	46.81	32.23
16	Downtown	Off	Enter	iOS	10	1.00	0.00	74.80	47.75
17	Downtown	Off	Enter	iOS	50	1.00	0.00	44.73	56.65
18	Downtown	Off	Enter	iOS	100	1.00	0.00	95.78	28.67
19	Downtown	Off	Exit	Android	10	0.40	0.55	10.52	NA
20	Downtown	Off	Exit	Android	50	0.80	0.45	117.45	60.33
21	Downtown	Off	Exit	Android	100	0.80	0.45	188.61	48.79
22	Downtown	Off	Exit	iOS	10	0.60	0.55	404.69	28.01
23	Downtown	Off	Exit	iOS	50	1.00	0.00	293.18	80.14
24	Downtown	Off	Exit	iOS	100	1.00	0.00	252.79	98.64
25	Forest	On	Enter	Android	10	0.00	0.00	NA	NA
26	Forest	On	Enter	Android	50	0.60	0.55	119.18	152.36
27	Forest	On	Enter	Android	100	0.80	0.45	30.68	33.48
28	Forest	On	Enter	iOS	10	1.00	0.00	81.45	60.90
29	Forest	On	Enter	iOS	50	1.00	0.00	76.27	48.59
30	Forest	On	Enter	iOS	100	1.00	0.00	82.05	68.14
31	Forest	On	Exit	Android	10	1.00	0.00	NA	NA
32	Forest	On	Exit	Android	50	1.00	0.00	332.64	NA
33	Forest	On	Exit	Android	100	1.00	0.00	456.77	NA
34	Forest	On	Exit	iOS	10	0.40	0.55	344.01	148.10
35	Forest	On	Exit	iOS	50	0.80	0.45	245.04	83.05
36	Forest	On	Exit	iOS	100	0.80	0.45	256.92	173.18
37	Forest	Off	Enter	Android	10	0.40	0.55	8.83	NA
38	Forest	Off	Enter	Android	50	0.40	0.55	254.17	NA
39	Forest	Off	Enter	Android	100	0.40	0.55	521.32	131.36
40	Forest	Off	Enter	iOS	10	1.00	0.00	98.88	64.12
41	Forest	Off	Enter	iOS	50	1.00	0.00	103.67	93.36
42	Forest	Off	Enter	iOS	100	1.00	0.00	100.50	47.43
43	Forest	Off	Exit	Android	10	1.00	0.00	NA	NA
44	Forest	Off	Exit	Android	50	1.00	0.00	NA	NA
45	Forest	Off	Exit	Android	100	1.00	0.00	585.55	NA
46	Forest	Off	Exit	iOS	10	0.40	0.55	321.24	109.97
47	Forest	Off	Exit	iOS	50	0.80	0.45	397.59	289.25
48	Forest	Off	Exit	iOS	100	0.80	0.45	438.21	122.04
49	Residential	On	Enter	Android	10	0.00	0.00	NA	NA
50	Residential	On	Enter	Android	50	1.00	0.00	25.53	18.10
51	Residential	On	Enter	Android	100	1.00	0.00	54.31	9.86
52	Residential	On	Enter	iOS	10	1.00	0.00	182.70	136.11
53	Residential	On	Enter	iOS	50	1.00	0.00	165.30	130.85
54	Residential	On	Enter	iOS	100	1.00	0.00	104.97	94.49
55	Residential	On	Exit	Android	10	0.20	0.45	34.89	NA
56	Residential	On	Exit	Android	50	0.80	0.45	102.50	28.46
57	Residential	On	Exit	Android	100	1.00	0.00	144.68	45.40
58	Residential	On	Exit	iOS	10	1.00	0.00	192.37	48.24
59	Residential	On	Exit	iOS	50	1.00	0.00	199.12	67.81
60	Residential	On	Exit	iOS	100	1.00	0.00	219.44	117.48
61	Residential	Off	Enter	Android	10	0.00	0.00	NA	NA
62	Residential	Off	Enter	Android	50	1.00	0.00	24.70	21.05
63	Residential	Off	Enter	Android	100	0.80	0.45	50.57	26.00
64	Residential	Off	Enter	iOS	10	1.00	0.00	136.70	45.81
65	Residential	Off	Enter	iOS	50	1.00	0.00	130.07	104.73
66	Residential	Off	Enter	iOS	100	1.00	0.00	61.61	39.61
67	Residential	Off	Exit	Android	10	0.40	0.55	90.36	56.42
68	Residential	Off	Exit	Android	50	0.80	0.45	107.29	14.73
69	Residential	Off	Exit	Android	100	1.00	0.00	165.28	38.59
70	Residential	Off	Exit	iOS	10	1.00	0.00	392.06	88.58
71	Residential	Off	Exit	iOS	50	1.00	0.00	533.01	223.36
72	Residential	Off	Exit	iOS	100	1.00	0.00	523.96	400.38

Each radius was repeated five times. The overall number of tests was 360 (3 areas x 2 Wi-Fi conditions x 2 types of events x 2 OS x 3 radii x 5 locations per radius). Sensitivity is defined as the probability of receiving a notification at a geofenced location (between 0 and 1). Distance is provided in meters between the location of the smartphone at the moment of receiving the notification and the center of the geofenced area

**Table 17:** Marginal effects of each experimental condition on sensitivity and distance to the center of the geofenced area in Study 1

		Enter		Exit	
		Sensitivity	Distance	Sensitivity	Distance
Radius	10 m	0.65 (0.48)	96.44 (85.54)	0.62 (0.49)	231.00 (146.23)
	50 m	0.90 (0.30)	81.79 (98.91)	0.92 (0.28)	227.72 (168.33)
	100 m	0.92 (0.28)	95.71 (99.97)	0.95 (9.22)	242.49 (152.09)
Environment	Forest	0.72 (0.45)	114.83 (129.16)	0.83 (0.38)	350.28 (168.98)
	Residential area	0.82 (0.39)	92.61 (91.90)	0.85 (0.36)	222.00 (170.45)
	Downtown	0.93 (0.25)	70.65 (75.75)	0.80 (0.40)	190.69 (102.35)
Wi-Fi	Off	0.81 (0.39)	85.75 (100.87)	0.82 (0.38)	293.25 (191.31)
	On	0.83 (0.37)	88.02 (91.90)	0.83 (0.37)	186.17 (98.98)
Operating system	Android	0.64 (0.48)	58.22 (104.04)	0.79 (0.41)	147.77 (103.46)
	iOS	1.00 (0.00)	104.31(86.20)	0.87 (0.34)	283.86 (160.62)

Mean (SD). As the enter and exit events represent substantially different experimental conditions affecting the geofencing, the marginal effects are presented separately for enter and exit events

**Table 18:** Marginal effects of operating system and radius on sensitivity and distance to the center of the geofenced area in Study 1

		Enter		Exit	
Operating system	Radius	Sensitivity	Distance	Sensitivity	Distance
Android	10 m	0.30 (0.47)	19.85 (8.97)	0.50 (0.51)	56.53 (51.83)
	50 m	0.80 (0.41)	46.46 (65.99)	0.90 (0.31)	114.22 (64.11)
	100 m	0.83 (0.38)	81.78 (140.43)	0.97 (0.18)	193.91 (116.91)
iOS	10 m	1.00 (0.00)	117.57 (85.14)	0.73 (0.45)	265.89 (133.31)
	50 m	1.00 (0.00)	106.86 (111.10)	0.93 (0.25)	298.16 (175.03)
	100 m	1.00 (0.00)	88.85 (51.02)	0.93 (0.25)	281.72 (167.46)

Mean (SD)

**Table 19:** Sensitivity, distance, and time difference with the fence in Study 2

	Event	OS	Radius (m)	Waiting 5 minutes in the center	Sensitivity (mean)	Sensitivity (SD)	Distance to the fence (mean)	Distance to the fence (SD)	Time difference (mean)	Time difference (SD)
1	Enter	Android	10	No	0.00	0.00	NA	NA	NA	NA
2	Enter	Android	10	Yes	0.80	0.45	14.52	15.12	81.00	56.08
3	Enter	Android	50	No	0.20	0.45	99.14	NA	116.00	NA
4	Enter	Android	50	Yes	1.00	0.00	40.46	17.76	242.20	268.00
5	Enter	Android	100	No	0.60	0.55	105.20	4.15	113.33	21.39
6	Enter	Android	100	Yes	1.00	0.00	56.03	43.54	126.60	126.95
7	Enter	Apple	10	No	1.00	0.00	-114.87	17.38	-119.20	29.75
8	Enter	Apple	10	Yes	1.00	0.00	-86.61	10.44	-103.80	14.60
9	Enter	Apple	50	No	1.00	0.00	-32.13	38.52	-35.80	38.07
10	Enter	Apple	50	Yes	1.00	0.00	-34.33	46.16	-56.20	64.64
11	Enter	Apple	100	No	0.80	0.45	40.67	38.26	35.75	27.84
12	Enter	Apple	100	Yes	1.00	0.00	-19.23	59.26	-34.60	81.86
13	Exit	Android	10	No	0.20	0.45	85.34	NA	97.00	NA
14	Exit	Android	10	Yes	0.00	0.00	NA	NA	NA	NA
15	Exit	Android	50	No	0.00	0.00	NA	NA	NA	NA
16	Exit	Android	50	Yes	0.60	0.55	126.95	38.51	193.67	128.06
17	Exit	Android	100	No	0.40	0.55	77.13	189.08	81.50	198.70
18	Exit	Android	100	Yes	1.00	0.00	128.78	44.41	264.40	191.28
19	Exit	Apple	10	No	0.40	0.55	175.68	13.21	187.00	49.50
20	Exit	Apple	10	Yes	0.60	0.55	174.20	27.21	250.67	79.73
21	Exit	Apple	50	No	1.00	0.00	140.91	31.36	165.40	66.82
22	Exit	Apple	50	Yes	0.80	0.45	169.76	30.63	226.00	35.19
23	Exit	Apple	100	No	0.80	0.45	93.50	46.89	107.50	49.70
24	Exit	Apple	100	Yes	1.00	0.00	109.81	33.75	119.20	47.47

Each radius was repeated five times. The overall number of tests was 120 (2 types of events x 2 OS x 3 radii x 2 types of user behavior x 5 locations per radius). Sensitivity is defined as the probability of receiving a notification at a geofenced location (between 0 and 1). Distance is provided in meters between the location where the fence was crossed and the location of the smartphone at the moment of receiving the notification. Time difference is provided in seconds between the moment of crossing the fence and receiving a notification

**Table 20:** Marginal effects of each experimental condition on sensitivity, distance, and time difference with the fence in Study 2

		Enter			Exit		
		Sensitivity	Distance	Time difference	Sensitivity	Distance	Time difference
Radius	10 m	0.70 (0.47)	-67.81 (57.03)	-56.50 (96.20)	0.30 (0.47)	159.89 (40.81)	203.83 (82.13)
	50 m	0.80 (0.41)	-1.93 (54.61)	54.19 (198.63)	0.60 (0.50)	147.22 (35.65)	192.67 (75.40)
	100 m	0.85 (0.37)	38.96 (60.01)	55.47 (103.67)	0.80 (0.41)	107.58 (63.09)	156.94 (138.63)
Operating system	Android	0.60 (0.498)	53.07 (39.57)	145.78 (159.27)	0.37 (0.49)	115.94 (69.64)	196.64 (166.75)
	iOS	0.97 (0.18)	-43.90 (60.25)	-55.34 (66.93)	0.77 (0.43)	139.60 (43.83)	168.83 (72.11)
User behavior	No waiting	0.60 (0.50)	-8.75 (87.77)	-9.78 (92.49)	0.47 (0.51)	119.25 (69.35)	135.07 (82.01)
	Waiting	0.97 (0.18)	-5.53 (60.25)	41.21 (172.34)	0.67 (0.48)	139.64 (41.36)	207.75 (118.61)

Mean (SD). As the enter and exit events represent substantially different experimental conditions affecting the geofencing, the marginal effects are presented separately for enter and exit events. Sensitivity is defined as the probability of receiving a notification at a geofenced location (between 0 and 1). Distance is provided in meters between the location where the fence was crossed and the location of the smartphone at the moment of receiving the notification. Time difference is provided in seconds between the moment of crossing the fence and receiving a notification

**Table 21:** Sensitivity and precision in Study 3

	Phone model	OS	OS Version	Mobile Internet Provider	Perceived mobile Internet quality	Enter event precision	Enter event sensitivity	Exit event precision	Exit event sensitivity
1	iPhone X	iOS	16.1.1	Blau	4	NA	NA	NA	NA
2	iPhone 13 mini	iOS	16.1.2	Telecom	3	0.80	1.00	NA	0.00
3	iPhone 11 Pro	iOS	16.0.2	Vodafone	3	0.81	1.00	0.75	0.80
4	Huawei P30 Pro	Android	12	Vodafone	3	0.13	0.60	1.00	0.20
5	iPhone 13	iOS	16.0.3	Vodafone	4	1.00	1.00	1.00	0.44
6	Samsung 8 FE	Android	13	Aldi Talk	3	1.00	0.38	NA	0.00
7	iPhone ?	iOS	16	Do not know	4	0.92	1.00	0.60	0.40
8	iPhone 11	iOS	16.1.2	Medionmedion	4	NA	0.00	NA	0.29
9	iPhone se	iOS	14.6	Vodafone	4	1.00	1.00	0.63	0.57
10	iPhone 13 Pro	iOS	16.1.1	Vodafone	4	0.87	1.00	1.00	0.50
11	iPhone 7	iOS		Medion	3	1.00	0.25	1.00	0.00
12	iPhone 12	iOS	15.6.1	Do not know	5	0.82	1.00	1.00	0.33
13	iPhone 6s	iOS	15.7.1	Vodafone	3	1.00	1.00	1.00	0.00
14	Huawei Mate 20 lite	Android	12	Vodafone	4	NA	0.00	NA	0.00
15	iPhone 11	iOS	16	Sim.de	4	1.00	0.67	1.00	0.33
16	SE2020	iOS	14.6	Medion	4	0.56	0.67	1.00	0.33
17	iPhone 11 Pro	iOS	16.0.3	o2	5	0.83	1.00	1.00	0.67
18	iPhone 7	iOS	15.7.1	Medion	4	1.00	0.88	1.00	0.25
19	iPhone 8	iOS	15.6.1	Medion	3	1.00	0.33	NA	0.00
20	iPhone 12	iOS	16.0.1	Telecom	4	NA	NA	NA	NA
21	iPhone 12	iOS	15.1	Telecom	4	0.00	1.00	NA	0.00
22	Oppo A16	Android	11	Mobile Com Debitel	3	0.30	0.78	1.00	0.11
23	Samsung Galaxy A50	Android		Blau	4	NA	NA	NA	NA
24	iPhone 11	iOS	16.1.1	Vodafone	5	0.83	0.57	1.00	0.29
25	Huawei P30 Pro	Android	12	o2	5	0.91	1.00	NA	0.00
26	Galaxy A70	Android	11	Do not know	3	0.86	0.89	NA	0.00
27	Huawei P40	Emui	12	Telecom	5	NA	NA	NA	NA
28	iPhone 8	iOS	15.6	o2	4	0.25	0.38	NA	0.00
29	iPhone 11	iOS	16	Do not know	3	1.00	0.50	1.00	0.00
30	Android	Android		Telecom	4	NA	0.00	NA	0.00
31	iPhone 8 Plus	iOS	14.7.1	Medion	2	0.82	1.00	1.00	0.11
32	iPhone 12	iOS	16.1.1	o2	4	1.00	0.83	1.00	0.17
33	Samsung A52s	Android	12	Telecom	5	NA	0.00	NA	0.00
34	Xiaomi ?	Android	12	Vodafone	4	0.64	0.86	0.80	0.57
35	iPhone 11 Pro	iOS	16.0.2	Vodafone	4	NA	0.00	NA	0.33
36	Samsung Galaxy A51	Android	12	1 & 1	3	0.45	1.00	1.00	0.17
37	iPhone XR	iOS	15.6.1	Congstar	4	0.00	0.14	NA	0.00
38	Xiaomi Redmi Note 10S	Android	12	Medion	4	0.22	0.89	NA	0.00
39	Huawei P20	Android	12	Medion	3	0.43	0.33	1.00	0.00
40	iPhone 7	iOS	15.6.1	AldiTalk	4	NA	0.88	NA	0.00
41	iPhone 8	iOS	16.1.1	Medion	4	0.47	0.78	1.00	0.22
42	Redmi Note 8T	Android	11	Vodafone	4	0.50	0.10	NA	0.00
43	iPhone 11	iOS	16.1	o2	4	0.60	0.90	1.00	0.30
44	iPhone 12	iOS	16.1.1	o2	4	1.00	0.00	NA	0.00
45	Doogee V20	Android	11	o2	4	0.77	1.00	1.00	0.14
46	iPhone 11	iOS	16.1.1	Medion	3	0.67	0.50	NA	0.00
47	iPhone 12	iOS	16	Vodafone	3	0.67	0.50	1.00	0.00
48	iPhone 11	iOS	16.0.2	o2	5	1.00	0.86	NA	0.00
49	iPhone 11	iOS	16.1.1	Medion	3	0.80	1.00	1.00	0.50
50	iPhone 11	iOS	16.1.1	Vodafone	3	0.33	1.00	NA	0.00
51	iPhone 13	iOS	16.1	Vodafone	3	0.94	1.00	1.00	0.29
52	Xiaomi 11t	Android	12	Medion	4	0.25	0.56	NA	0.00
53	iPhone 8	iOS	16.0.3	Medion	4	0.50	0.25	NA	0.00
54	iPhone SE	iOS	16.1.1	Vodafone	4	1.00	1.00	1.00	0.25
55	Galaxy A52	Android	12	Telecom	3	0.71	0.83	NA	0.00
56	iPhone X	iOS	16.1.1	Do not know	5	0.83	0.50	0.50	0.00
57	iPhone 12 mini	iOS	16.0.2	Freenet	4	0.89	1.00	1.00	0.40
58	iPhone XR	iOS	16.1.1	Medion	3	0.67	0.88	0.50	0.13

The perceived quality of mobile Internet connectivity was measured on a five-point Likert scale ranging from “very poor” (1) to “excellent” (5). Question marks in the name of a phone model (e.g., "Xiaomi ?") mean that the participant has not provided information about the specific model of the phone

**Table 22:** Statistical power estimates

	Sensitivity			Distance to the center	Distance to the fence	Time difference with the fence	Precision
	Study 1	Study 2	Study 3	Study 1	Study 2	Study 2	Study 3
Forest (vs. downtown)	53%	-	-	92%	-	-	-
Residential area (vs. downtown)	26%	-	-	42%	-	-	-
Wi-Fi is on (vs. off)	18%	-	-	86%	-	-	-
Exit event (vs. enter)	17%	79%	100%	100%	100%	100%	51%
iOS (vs. Android)	100%	97%	19%	99%	77%	95%	18%
50 50-m radius (vs. 10 m)	99%	57%	-	19%	62%	47%	-
100 100-m radius (vs. 10 m)	99%	89%	-	26%	59%	23%	-
Waiting 5 min (vs. no waiting)	-	93%	-	-	18%	32%	-

We used a simulation approach in which model parameters were used to simulate the outcome variable (with 1000 repetitions). After that, a model was fit into the simulated data, and the effects were estimated. The percentage indicates the number of cases in which the effect turned out to be statistically significant (below the alpha level of 0.05).

## Footnotes

1 The mobile application meets the data security requirements of both Android and iOS application stores, and is revised periodically to comply with new updates related to data security and confidentiality. The data are stored on the server of the University of Konstanz in Germany. For more information, please see the Terms of Use and Privacy Policy at https://samply.uni-konstanz.de/.

2 The general documentation is available at the *Samply* website https://samply.uni-konstanz.de/docs/intro and the information on geofencing can be found in the *Samply* tutorial (linked at https://samply.uni-konstanz.de/).

3 iOS and Android accounted together for 99.23% of market share worldwide in January 2022 according to StatCounter Global Stats (retrieved on February 23, 2022) https://gs.statcounter.com/os-market-share/mobile/worldwide. The description of how iOS and Android handle geofencing is accurate at the time of writing, but it will be subject to change in the future. Researchers should consult the latest version of the developer documentation for iOS and Android.

4 Apple developer documentation https://developer.apple.com/videos/play/wwdc2020/10660/

5 Region monitoring in iOS https://developer.apple.com/documentation/corelocation/monitoring_the_user_s_proximity_to_geographic_regions

6 Android developer documentation https://developer.android.com/training/location/geofencing#choose-the-optimal-radius-for-your-geofence

7 Beginning with Android 4.3, there is a “Wi-Fi scan only mode” that allows users to disable Wi-Fi but still get the network location, which is sufficient for geofencing.

8 The absolute geolocation was recorded inside of the web survey accessed through the smartphone’s mobile browser. Because of the poor Internet connection, this survey could not be open, and therefore the location was not recorded.

9 The percentage was calculated as the proportion of locations where a message was received at least once, *n* = 297. In 17 cases, a notification for the same location was sent multiple times by the server. These repetitions were not included in the analysis.

10 In post hoc assessments of the forest area, we found that sites 106–111 did not have Internet connectivity, which was indicated by zero connection bars in the smartphone menu.

11 We thank an anonymous reviewer who suggested to include the condition of staying inside the fenced area and record the time of notification.

12 https://developer.apple.com/library/archive/documentation/UserExperience/Conceptual/LocationAwarenessPG/RegionMonitoring/RegionMonitoring.html#//apple_ref/doc/uid/TP40009497-CH9-SW11

13 The documentation on the doze mode in Android: https://developer.android.com/training/monitoring-device-state/doze-standby

14 Recommendations for adjusting settings on Android devices to ensure that geofencing remains functional: https://dontkillmyapp.com/
